# Characteristics of Films Prepared from Wheat Gluten and Phenolic Extracts from *Porphyra haitanensis* and Its Application for Salmon Preservation

**DOI:** 10.3390/foods13152442

**Published:** 2024-08-02

**Authors:** Tingyue Yu, Jingwen Xu

**Affiliations:** College of Food Science, Shanghai Ocean University, Shanghai 201306, China; yutingyue0308@163.com

**Keywords:** wheat gluten, phenolic extracts, *Porphyra haitanensis*, storage qualities, shelf life

## Abstract

The effect of wheat gluten (WG)/phenolic extracts (PE) coating on the storage qualities of salmon fillets was studied. *Porphyra haitanensis*, belonging to red algae, possesses abundant phenolic compounds. Films were prepared by incorporating phenolic extracts (0, 0.5%, 0.75%, and 1.0%, *w*/*v*) from *Porphyra haitanensis* to WG. The PE showed strong antioxidant activities by scavenging DPPH and ABTS radicals. The increased addition of PE to WG film significantly increased tensile strength compared to that of WG film, but reduced water vapor permeability. The quality of salmon fillet stored at 4 °C from 0 to 9 days was decreased due to the oxidation of lipid and protein. However, the increased addition of PE to WG significantly reduced pH, TVB-N, TBA, peroxide value, total sulfhydryl content, and carbonyl content of salmon fillet compared to control salmon fillet. In addition, the increased addition of PE to WG also significantly improved water holding capacity, hardness, chewiness, and springiness of salmon fillet during storage compared to those of control salmon fillet. Taken together, this study showed phenolic extracts from *Porphyra haitanensis* improved wheat gluten-based film properties and further enhanced the qualities of coated salmon fillet during storage.

## 1. Introduction

Food packaging plays an important role in protecting foodstuffs from physical, chemical, and biological contamination effectively for maintaining the safety and quality of foodstuffs, as well as extending the shelf life. Traditionally, food packaging mainly relies on petroleum-based polymers. Recently, biodegradable polymers have been increasingly studied and substituted to petroleum-based polymers for reducing environmental pollution. Currently, biodegradable polymers used in food packaging are mainly divided into two classifications including native polymers such as starch, protein, cellulose, chitin, etc., and synthetic polymers such as polylactic acid (PLA), polybutylene succinate (PBS), etc. [1,2,3,4]. Native polymers, such as wheat gluten, have recently been attracting increased attention in food packaging applications due to its biodegradability, low cost, edibility, and good oxygen barrier property [5,6]. However, pure wheat gluten film is limited by its poor water resistance and mechanical characteristics due to the hydrophilic nature. Therefore, wheat gluten-based film is usually strengthened by adding other strengthened compounds such as polysaccharides, proteins, lipids, nanofillers, and phenolic compounds [5].

An increasing number of studies have been focused on food packaging containing phenolic compounds for maintaining food quality and extending shelf life [7]. Previously, we have identified the phenolic profile of “blue food” *Porphyra haitanensis* and found the strong antioxidant activities of phenolic extracts [8], which was considered to potentially prevent the lipid oxidation of fish muscles. However, to date, there is no available study yet about the effect of phenolic extracts from *Porphyra haitanensis* on the storage qualities of salmon fillets.

Therefore, the aim of this study was to incorporate antioxidative phenolic extracts from *Porphyra haitanensis* to wheat gluten-based film and further investigate the effect of phenolic extracts on film properties including physical, mechanical, and water barrier properties. The effects of the WG/PE coating on salmon fillet stored at 4 °C from 0 to 9 days in terms of pH, TVB-N, TBA, peroxide value, water holding capacity, texture, total sulfhydryl content, and carbonyl content will be evaluated. This study will help better understand the effects of phenolic extracts from *Porphyra haitanensis* on the characteristics of wheat gluten-based films and their effects on the qualities of salmon fillet during storage.

## 2. Materials and Methods

### 2.1. Materials

*Porphyra haitanensis* was bought from XiaPuCongHui Trading Co., Ltd. (Fuding City, Fujian Province, China). Wheat gluten was purchased from Jianlong Bio-Tech Co., Ltd. (Hohot, Inna Mongolia, China). Salmon was purchased from Kunshan Xielaoshan Co., Ltd. (Kunshan, Jiangsu Province, China). Purity of wheat gluten is over 80%. Ethanol was purchased from Shanghai Sangon Biotech Co., Ltd. (Shanghai, China). Chemicals including glycerol, sodium chloride, boric acid, and magnesium oxide were purchased from Shanghai Macklin Co., Ltd. (Shanghai, China). Reagents including methylene blue, bromocresol green, potassium chloride, and sodium chloride were bought from Shanghai Yuanye Biology Co., Ltd. (Shanghai, China). Total sulfhydryl content kit and total carbonyl content kit were purchased from Nanjing Jiancheng Biology Engineering Institute (Nanjing, China). All chemicals used in this study were at analytical level.

### 2.2. Phenolic Compounds Extraction from Porphyra haitanensis and Characterization of Antioxidant Activities

Extraction of phenolic compounds and antioxidant activities by scavenging DPPH and ABTS were followed by our previous method [8].

### 2.3. Wheat Gluten/Phenolic Extracts-Based Film Preparation

Wheat gluten/phenolic extracts (WG/PE)-based film was prepared according to Chen et al. (2022) with modifications [9]. Ten grams of wheat gluten was added to 100 mL of DI water and stirred at 25 °C for 10 min. Then, the solution was heated to 90 °C for 5 min. Glycerol (30%, on protein basis, dry basis) was added to the wheat gluten solution. Phenolic extracts were added to the solution at 0%, 0.5%, 0.75%, and 1%, (*w*/*v*) on the solution basis. After cooling down, 10 mL of the solution was collected and poured into a Petri dish (90 mm diameter) and dried in an oven at 40 °C for solution evaporation and forming composite film.

### 2.4. Characterization of Film

#### 2.4.1. Color Measurement and Opacity Value

The color was measured using a colorimeter (Colorimeter, Model TS8216, Guangdong 3nh Technology Co., Ltd., Guangzhou, China). The films were cut into 5 cm (l) × 2 cm (w) for the measurement. The instrument was calibrated against a white tile. Data including lightness (L*), redness/greenness (a*), yellowness/blueness (b*), and total color difference (ΔE) were collected and calculated. Measurements were conducted directly on the film surface. Each test was repeated for five replicates.

$$\Delta E=\sqrt{{\left(\Delta L^{*}\right)}^{2}+{\left(\Delta a^{*}\right)}^{2}+{\left(\Delta b^{*}\right)}^{2}}$$

Wherein, ∆L*, ∆a* and ∆b* were the difference between the sample and reference, respectively.

The opacity value of the films was measured at the ultraviolet and visible range of 200–800 nm using a UV-vis spectrophotometer (UV-3600, Japan Mitutoyo Co., Ltd., Tokyo, Japan). Each film was cut into 20 mm (l) × 5 mm (w) for the test. The opacity value of the film was calculated according to the following equation:

$$\mathrm{OV}=\frac{-LogT600}{X}$$

where T600 was the fractional transmittance at 600 nm and *x* was the film thickness (mm). Each test was repeated for five replicates.

#### 2.4.2. Thickness

The thickness of film was determined using a micrometer (Micrometer, Model 200 mm 500–196, Japan Mitutoyo Co., Ltd., Tokyo, Japan). Each film was measured for ten replicates.

#### 2.4.3. Water Vapor Permeability (WVP)

The WVP of films was measured following the method of Liang et al. (2018) with modifications [10]. The films with 10 mm diameter were cut and filled with 200 mL DI water in a beaker, which was sealed with paraffin. The test was conducted at 25 °C and 75% relative humidity (RH). The weight change in film was measured by a microbalance every 2 h up to 12 h. The water vapor transmission rate (WVTR) was calculated from the slope of the weight change versus time. The WVP was calculated using the following equations:

$$\mathrm{WVTR}\;(g\cdot m^{-2}\cdot d^{-1})=\frac{\Delta W}{A\times \Delta t}$$

where Δ*W* was the mass of water vapor passing through the film (g), *A* was the area of the film (cm^−2^), and Δ*t* was the time interval (day).

$$\mathrm{WVP}\;(g\cdot mm\cdot m^{-2}\cdot {\mathrm{day}}^{-1}\cdot {\mathrm{kPa}}^{-1})=\frac{WVTR\cdot Z\cdot 100}{Ps\cdot RH\left(100\%\right)}$$

Wherein, Z was the film thickness (mm), *Ps* was the water vapor saturation pressure at test temperature (kPa), and *RH (%)* was the percentage relative humidity gradient.

Each test was repeated for three replicates.

#### 2.4.4. Tensile Strength (TS)

The TS of the films was determined using a Tensile Testing Machine (Automatic tensile testing machine, Model XLW-EC, Ji’an Opto-Electro-Mechanical Technology Co., Ltd., Ji’an, China) according to the previous method with modifications [7]. The tested films were cut into 15 mm (l) × 100 mm (w). The initial grip separation and mechanical crosshead speed were set as 65 mm and 50 mm/min until break.

$$\mathrm{TS}\;(\mathrm{MPa})=\frac{F}{A}$$

Wherein, *F* (N) was the peak force at failure and *A* (mm^2^) was the cross-sectional area of films.

Each test was repeated for three replicates.

#### 2.4.5. FTIR

The films were analyzed using a FTIR spectrophotometer (FTIR, Tianjin Gangdong Sci. & Tech. Development Co., Ltd., Tianjin, China). The films were freeze-dried, milled, and sieved through 80 mesh before testing. Films were mixed with KBr at the ratio of 1:100. The pure KBr was used as the reference. The region of spectra was from 400 to 4000 cm^−1^ with the resolution of 4 cm^−1^ and was scanned 32 times.

### 2.5. Quality Changes in Salmon Fillet Packed with WG/PE

#### 2.5.1. Preparation and Storage of Salmon Fillet

Salmon was cut into 3 cm (l) × 10 cm (w) × 5 cm (h) and soaked in WG/PE solution for 1 min, then waited for evaporation of WG/PE solution for 15 min. WG/PE-coated salmon were covered with sterile bags and placed at 4 °C for storage. Following tests were measured at days of 0, 3, 6, and 9.

#### 2.5.2. pH

Two grams of salmon was weighed and mixed with 20 mL of DI water, homogenized, and tested by pH meter (pH meter, Model PHSJ-4F, Shanghai Leici Experimental Instrument Co., Ltd., Shanghai, China). Each test was repeated for three replicates.

#### 2.5.3. Total Volatile Basic Nitrogen (TVB-N)

The determination of TVB-N was followed the Chinese National Standard of GB 5009.228-2016 [11]. The result was expressed as mg TBV-N/100 g of the sample. Each test was repeated for three replicates.

#### 2.5.4. Thiobarbituric Acid (TBA)

The determination of TBA followed Niu et al. (2022) with modifications [12]. Ten grams of salmon was mixed with 25 mL of DI water and homogenized for 2 min (Homogenizer, Bagmixer, Shanghai Zhikeyi Co., Ltd., Shanghai, China). Twenty-five mL of 5% (*m*/*v*) trichloroacetic acid was added, mixed well and left for 30 min. Five mL of supernatant was selected and added with 5 mL of 0.02 mol/L thiobarbituric acid solution in a water bath at 95 °C for 40 min. After cooling to room temperature, the resultant solution was assayed by a UV-vis spectrophotometer at 532 nm (UV-3600, Japan Mitutoyo Co., Ltd., Tokyo, Japan). The result was expressed as the mass fraction of MDA in mg/kg.

$$\mathrm{TBA}=\frac{As-Ab}{100}$$

Wherein, *As* was the absorbance of sample against water blank and *Ab* was the absorbance of reagent blank. Each test was repeated for three replicates.

#### 2.5.5. Estimation of Peroxide Value (PV)

The PV of salmon was described by Zhang et al. (2021) with modifications [13]. Ten grams of salmon was mixed well with 30 mL of petroleum ether. The solution was extracted for 24 h before filtering and removing the solvent by rotary evaporation. An aliquot of 3 g of filtrate and 1 mL potassium iodide solution with 60% (*v*/*v*) acetic acid/chloroform solution (30 mL) was mixed for 3 min in the dark. A total of 100 mL DI water was added, and the solution was titrated with 0.01 M Na_2_S_2_O_3_ containing starch-iodide indicator to an equivalence point. A reagent blank was prepared in the same way without salmon. The PV was calculated as the following equation:

$$\mathrm{PV}\;(\mathrm{meq}/\mathrm{kg})=\frac{V-V_{0}}{m}\times 100$$

Wherein, *m* was the salmon weight (g), *V* and *V_0_* were the volume of Na_2_S_2_O_3_ used for salmon and blank (mL), respectively. The PV result was reported as milliequivalents (meq) of active oxygen per kg of lipid. Each test was repeated for three replicates.

#### 2.5.6. Water Holding Capacity (WHC)

Five grams of salmon was weighed, grinded and solubilized in a 70 °C water bath for 20 min. Then, samples were centrifuged at 5000 rpm for 5 min (TD25-WS, Suqian Huazhiheng E-commerce Co., Ltd., Suqian, China). Water in the salmon was soaked and weighed, and repeated for three times.

$$\mathrm{Water}\;\mathrm{holding}\;\mathrm{capacity}\;(\%)=\frac{W_{2}}{W_{1}}\times 100\%$$

Wherein, *W*_1_ and *W*_2_ was the weight before and after centrifugation (g), respectively.

Each test was repeated for three replicates.

#### 2.5.7. Determination of Total Sulfhydryl Content

Salmon of 0.1 g were weighed and grinded in ice and tested using a sulfhydryl content kit. The standard curve was made using 25 μmol/mL of sulfhydryl standard solution diluted to 0.5, 0.25, 0.125, 0.0625, 0.03125, and 0.015625 μmol/mL.

Standard curve: Y = 1.6378x + 0.1062 (R^2^ = 0.9991)

where y was the concentration (µmol/mL) and x was the absorbance at 412 nm.

Total sulfhydryl content: (μmol/g) = $\frac{y\;\times \;V}{W}$

where w was the weight of salmon. Each test was repeated for three replicates.

#### 2.5.8. Determination of Carbonyl Content

Salmon of 0.1 g were weighed and grinded in ice and tested using a carbonyl content kit. The equation of carbonyl content was the following:

$$\mathrm{Carbonyl}\;\mathrm{content}\;(\mu \mathrm{mol}/g)\;\frac{A_{\mathrm{sample}}-A_{\mathrm{control}}}{\varepsilon \times d}\times V\times \frac{W\times V_{\mathrm{sample}}}{V_{solution}}=\frac{A_{sample}-A_{control}}{8\times W}$$

where ε was the protein carbonyl extinction coefficient, equaled to 22 mL/μmol/cm; d was the cuvette light path, equaled to 1 cm; V was the volume of added solution (=1 mL); V_sample_ was the volume of sample (=0.8 mL); V_solution_ was the volume of added solution (=2.2 mL); and W was the weight of sample (=0.1 g). Each test was repeated for three replicates.

#### 2.5.9. Texture Analysis

Textural profile analysis was performed using a Texture Analyzer (SMS TA. XT Plus analyzer, Stable Micro System, Godalming, UK) according to previous method [14]. Hardness, chewiness, and springiness were calculated. Each treatment was measured for five replicates.

### 2.6. Statistical Analysis

Statistical test was performed using SAS. 9.4 software (SAS Ins., Cary, NC, USA). Statistical difference was compared through Tukey’s test (α ≤ 0.5).

## 3. Results

### 3.1. Antioxidant Activities of Phenolic Extracts

**Figure 1:**
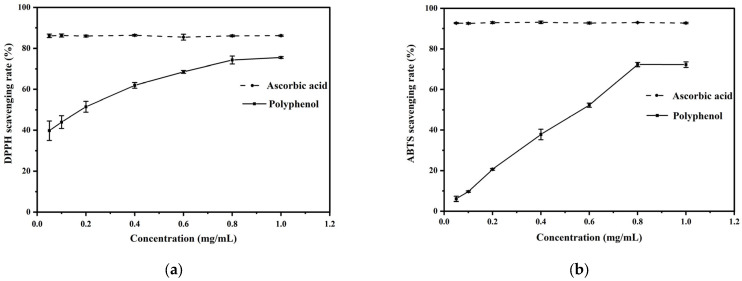
Antioxidant activities of phenolic extracts. DPPH scavenging rate (**a**) and ABTS scavenging rate (**b**).

The antioxidant activities of phenolic extracts were evaluated by scavenging DPPH and ABTS free radicals (Figure 1). Phenolic extracts showed a dose-dependent effect, that the increased concentration of phenolic extracts resulted in the increased scavenging rate of DPPH and ABTS free radicals. An amount of 1 mg/mL of phenolic extracts showed a DPPH and ABTS scavenging rate around 70% and 72%, respectively.

### 3.2. Film Properties

#### 3.2.1. Color Measurement and Opacity Value

**Figure 2:**
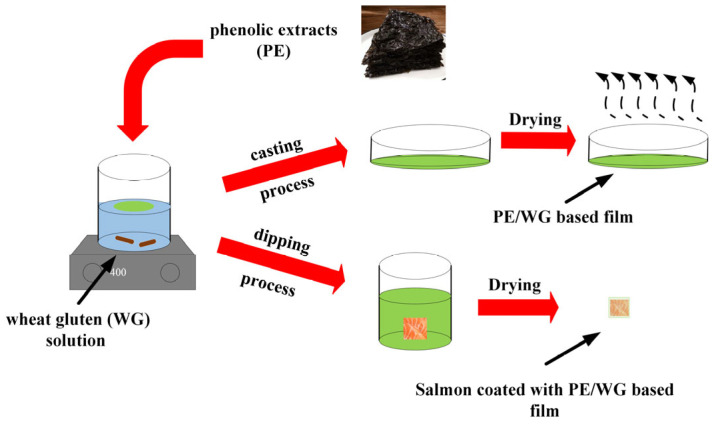
Schematic diagram of synthesis of WG/PE coating.

The schematic diagram of synthesis of the WG/PE coating is shown in Figure 2. The color measurement and opacity value of films are shown in Table 1. The transparency and color of foods affect consumer acceptance, as they can directly affect the appearance of the product. The addition of PE of 0.5–1% to WG resulted in a significant decrease in the L*, a*, b*, ΔE, and opacity of films (*p* ≤ 0.05), indicating that films turned darker. This was in an agreement with previous studies [15,16].

#### 3.2.2. Thickness, WVP, and TS

The thickness, WVP, and TS of films are shown in Table 2. The addition of PE to WG resulted in a significant decrease in thickness (0.22–0.18 mm) (*p* ≤ 0.05) and a significant increase in TS (6.70–8.30 Mpa) of films (*p* ≤ 0.05). The addition of 0.5% PE to WG significantly increased WVP of films compared to WG film (*p* ≤ 0.05). The addition of 0.75–1.0% of PE to WG further significantly decreased the WVP of films in contrast to WG film (*p* ≤ 0.05).

#### 3.2.3. FTIR Analysis

**Figure 3:**
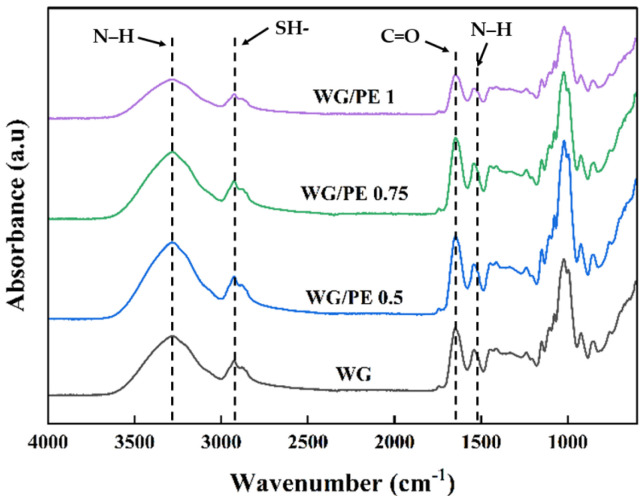
FTIR spectra of films.

The FTIR analysis of the WG/PE films is shown in Figure 3. The strong absorbance at 3300 cm^−1^ was assigned to free hydroxyl groups and amine N–H stretching [17]. The peaks at 1630, 1532, and 1235 cm^−1^ were due to the C = O stretching vibration of amide I, N–H blending of amide II, and C–N stretching of amide III, respectively, and belonged to the secondary structure of wheat gluten [18]. The peak at 1630 cm^−1^ slightly shifted to the left, indicating the interactions between WG and PE [19]. The absorbance at 2928 cm^−1^ was due to the asymmetric stretch vibrations of C–H and –NH^3+^ [20]. The peaks at 2830 and 495–635 cm^−1^ were due to the SH- and S-S band, respectively [21]. The increased addition of PE to WG reduced the intensity of the peak at 3300 cm^−1^, indicating the increased energy required for the vibration of functional groups and functional groups were more stable and compatible. In addition, the band of 3100–3700 cm^−1^ of WG/PE films turned to be broader as the addition of PE increased, indicating the formation of a hydrogen bond between WG and PE [19].

### 3.3. WG/PE on Salmon Fillet

#### 3.3.1. pH

**Figure 4:**
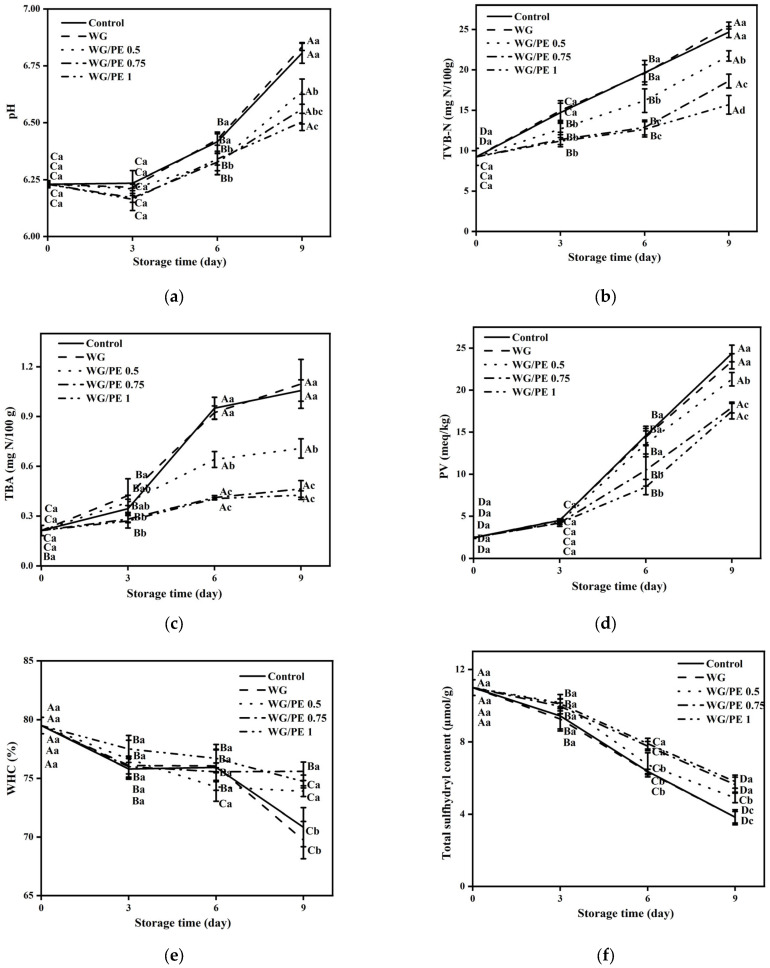

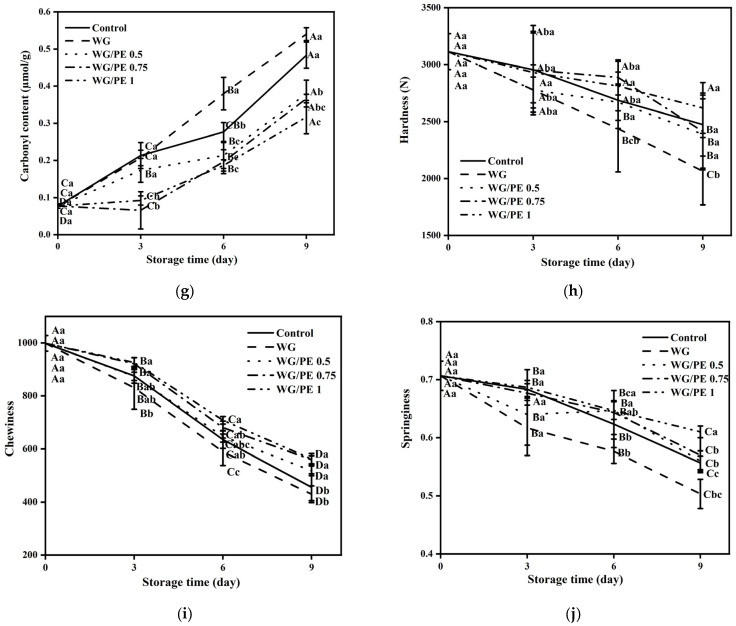
Effect of WG/PE coating on pH (**a**), TVB-N (**b**), TBA (**c**), PV (**d**), WHC (**e**), total sulfhydryl content (**f**), carbonyl content (**g**), hardness (**h**), chewiness (**i**), and springiness (**j**). N = 3. Values are expressed as ave ± stdev. Different uppercase letters compared within the same coating during storage 0–9 days indicated significant difference (*p* ≤ 0.05). Different lowercase letters compared within the same storage day with different coating indicated significant difference (*p* ≤ 0.05).

The pH of salmon fillet stored at 4 °C from 0 to 9 days was shown in Figure 4a. The change in pH is an important indicator for evaluating the spoilage of fishery products. The pH of fresh salmon initially at day 0 was 6.23, which was considered normal since the pH usually varies from 6.1 to 6.3 [22]. There was no significant change in pH at storage of 0–3 days, whereas the pH significantly increased from 3 to 9 days (*p* ≤ 0.05).

#### 3.3.2. TVB-N

The TVB-N of salmon fillet stored at 4 °C from 0 to 9 days is shown in Figure 4b. TVB-N is commonly used for evaluating the freshness of fishery products due to indicating the spoilage of production of trimethylamine (TMA) [23]. The change in TVB-N is highly correlated to fish autolysis by the proteinase enzyme degradation of muscle into ammonia (NH_3_), amines, dimethylamine (DMA), and trimethylamine (TMA) [12]. The value of TVB-N of salmon fillet was increased significantly as the storage time increased from 3 to 9 days (*p* ≤ 0.05).

#### 3.3.3. Thiobarbituric Acid (TBA)

The thiobarbituric acid (TBA) of salmon fillet stored at 4 °C from 0 to 9 days is shown in Figure 4c. Lipid oxidation is a chain reaction during storage. The thiobarbituric acid reactive substances (TBARS) value can be used to determine the secondary oxidation products, mainly generated from the decomposition of peroxides to aldehydes and ketones [24]. Therefore, TBA is an important indicator of freshness of fish muscle. The TBA significantly increased as the storage time increased from 0 to 9 days (*p* ≤ 0.05). At the same storage time of 3–9 days, the increased addition of PE to WG significantly reduced TBA (*p* ≤ 0.05) compared to the control treatment and salmon coated with WG, indicating that the PE addition inhibits the lipid oxidation of salmon fillet. Phenolics were reported to possess antioxidant activities against endogenous enzymes, thus impeding the lipid reaction [25].

#### 3.3.4. PV

The PV of salmon fillet stored at 4 °C from 0 to 9 days is shown in Figure 4d. The PV is usually used for evaluating the concentration of hydroperoxides formed at the initial stage of lipid oxidation [26]. The PV significantly increased as the storage time increased from 3 to 9 days (*p* ≤ 0.05), whereas the increased addition of PE to WG significantly reduced the PV at day 6 and 9 (*p* ≤ 0.05).

#### 3.3.5. WHC

The WHC of salmon fillet stored at 4 °C from 0 to 9 days is shown in Figure 4e. The WHC of salmon fillet significantly decreased during storage from 0 to 9 days (*p* ≤ 0.05). Cao et al. (2021) reported that the WHC was mainly influenced by factors including the destruction of the muscle fiber structure, myosin denaturation, and change in extracellular space [27]. The reduced WHC of salmon fillet during storage was probably attributed to the increased gaps between the initial tightly arranged myofibrils induced by proteolytic activity of enzymes and protein degradation as the storage days increased [28]. In addition, the increased addition of PE to WG resulted in the significant increase in WHC of salmon fillet at day 9 (*p* ≤ 0.05), indicating that the hydrophilicity of PE prevented the moisture loss during storage.

#### 3.3.6. Total Sulfhydryl Content

The total sulfhydryl content of salmon fillet stored at 4 °C from 0 to 9 days is shown in Figure 4f. A significant decrease in the total sulfhydryl content of salmon fillet was observed during storage from 3 to 9 days (*p* ≤ 0.05). This finding was in accordance with previous studies [12,29]. The total sulfhydryl content of salmon fillet can be used to evaluate the protein oxidation during storage, and the low content of total sulfhydryl indicates the high oxidation level of protein. Sulfhydryl groups were mainly present in the myofibrillar proteins and divided into two classifications including those exposed at the surface and those buried in proteins [30]. The decrease in total sulfhydryl content of salmon fillet during storage was associated with the oxidation of sulfhydryl groups in the myofibrillar protein and the formation of disulfide bonds [29]. In addition, the unfolding of myofibrils protein resulted in the exposure of hidden sulfhydryl groups at the surface of protein and under the oxidative conditions, and therefore resulted in the reduction in the total sulfhydryl content [31].

#### 3.3.7. Carbonyl Content

The carbonyl content of salmon fillet stored at 4 °C from 0–9 days is shown in Figure 4g. The carbonyl group of proteins is a major product of protein oxidation. Oxidation resulted in the changes in the structure and functions of myofibrillar proteins such as fragmentation, crosslinking, and aggregation, as well as the transformation of amino acid residues as lysine, arginine, protein, and histidine residue to form carbonyl compounds [31]. The carbonyl content of salmon fillet significantly increased as the storage time increased from 3 to 9 days (*p* ≤ 0.05), which was in agreement with Zhao et al. (2019) [32]. The increased addition of PE to WG significantly reduced the carbonyl content of salmon fillet from day 3–9 compared to those of the control treatment and WG-coated salmon fillet, indicating the inhibitory effect of PE against the oxidation of fish muscle, which was in accordance with Nuerjiang et al. (2023) [33].

#### 3.3.8. Textural Profile Analysis

The textural profile of salmon fillet including hardness, chewiness, and springiness at 4 °C from 0 to 9 days is shown in Figure 4h–j. The hardness, chewiness, and springiness of salmon fillet significantly decreased as the storage time increased from 0 to 9 days (*p* ≤ 0.05). There was no significant difference in the hardness of the salmon fillet between the control treatment and the those coated with WG or WG/PE during storage of 0–6 days. However, at storage day 9, the addition of PE to WG coating significantly increased the hardness of the salmon fillet compared to the control treatment and WG-coated salmon (*p* ≤ 0.05). Chewiness is an important indicator reflecting the freshness and organoleptic quality of fish muscle. The increased addition of PE to WG significantly increased the chewiness of salmon fillet during storage of 3–9 days compared to the control treatment and WG-coated salmon fillet (*p* ≤ 0.05). The decrease in the chewiness of fish muscle was attributed to the lipid oxidation, resulting in the myofilament degradation and costamere distraction [34]. The reduced springiness of the salmon fillet as the storage time increased from 0 to 9 days (*p* ≤ 0.05) was in line with Wang et al. (2021) [25].

## 4. Discussion

The increased pH of salmon fillet during storage of 3–9 days was mainly attributed to the formation of nitrogenous compounds such as ammonia, trimethylammonium, histamine, etc., which were formed from the autolysis of endogenous enzymes and microbial enzymatic action [31]. The increased addition of PE to WG significantly reduced the TVB-N of salmon fillet compared to the control treatment and salmon coated with WG (*p* ≤ 0.05) during storage of 3–9 days. This was in agreement with previous studies, which showed that phenolic extracts reduced the TVB-N value and improved the freshness of fish muscle [23,35].

In our study, the initial TVB-N of salmon fillet was around 8 mg N/100 g, indicating the high freshness [35]. According to the national standard of China of GB/T18108-2019 [36], TVB-N < 15 mg/100 g was considered to be premium product; 15 mg/100 g < TVB-N < 30 mg/100 g was considered to be qualified product. We found that, at the storage time of 9 day, the films of WG/PE 0.5, WG/PE 0.75, and WG/PE 1.0 maintained salmon fillet qualified products.

The oxidation of protein and lipid was initially generated by the reactive oxygen species, and the intermediate compounds generated during oxidation could further accelerate oxidation chain reactions and impact each other [23]. Phenolic compounds from *Porphyra haitanensis* showed strong antioxidant activities against lipid oxidation; thus, the increased addition of PE to WG films further reduced the PV of salmon fillet.

The total sulfhydryl content of salmon fillet can be used to evaluate the protein oxidation during storage, and the low content of total sulfhydryl indicates the high oxidation level of protein. Sulfhydryl groups were mainly present in the myofibrillar proteins and divided into two classifications, including those exposed at the surface and those buried in proteins [30]. The decrease in the total sulfhydryl content of salmon fillet during storage was associated with the oxidation of sulfhydryl groups in the myofibrillar protein and the formation of disulfide bonds [29]. In addition, the unfolding of myofibrils protein resulted in the exposure of hidden sulfhydryl groups at the surface of protein and under the oxidative conditions, and therefore resulted in the reduction in total sulfhydryl content [31].

Christensen et al. (2017) reported that the decreased hardness of fish muscle during storage was correlated to the WHC and degradation of structural proteins by proteolytic enzymes [37]. In our findings, the increased addition of PE to WG improved the WHC of salmon fillet at day 9 significantly compared to other treatments (*p* ≤ 0.05), which was in accordance with Christensen et al. (2017) [37]. The decrease in the chewiness of fish muscle was attributed to the lipid oxidation, resulting in the myofilament degradation and costamere distraction [34]. The reduced springiness of the salmon fillet as the storage time increased from 0 to 9 days (*p* ≤ 0.05) was in line with Wang et al. (2021) [25].

## 5. Conclusions

In this study, films prepared from wheat gluten and phenolic extracts from *Porphyra haitanensis* were characterized and their application on salmon fillet stored at 4 °C during 0–9 days was also evaluated. This was the first study to incorporate the phenolic extracts from *Porphyra haitanensis* to wheat gluten-based film and analyze their effect on the film properties and coated salmon fillet during storage. The increased addition of PE from 0.50 to 1.00% (*w*/*v*) to WG significantly increased the water vapor permeability and tensile strength of resultant films. The effects of WG/PE coating on salmon fillet stored at 4 °C from 0 to 9 days were also characterized. The WG/PE coatings impacted pH, TVB-N, TBA, PV, WHC, texture, total sulfhydryl content, and carbonyl content of salmon fillet at different extents during storage. In summary, the incorporation of PE from *Porphyra haitanensis* to WG films improved the film properties and qualities of coated salmon fillet during storage.

## Figures and Tables

**Table 1:** Color measurement of coatings.

Treatment	L*	a*	b*	ΔE	Opacity Value
WG	54.62 ± 0.72 a	−0.19 ± 0.04 a	10.92 ± 0.84 a	55.71 ± 0.66 a	3.22 ± 0.30 a
WG/PE 0.5	51.81 ± 1.45 b	−0.61 ± 0.02 c	10.34 ± 0.29 ab	52.83 ± 1.48 b	2.53 ± 0.15 bc
WG/PE 0.75	48.29 ± 2.59 bc	−0.50 ± 0.07 b	9.56 ± 0.80 bc	49.29 ± 2.57 c	2.26 ± 0.09 c
WG/PE 1	44.40 ± 0.94 d	−0.53 ± 0.02 bc	8.82 ± 0.35 c	45.27 ± 0.98 d	2.21 ± 0.08 c

N = 5. Values are expressed as ave ± stdev. Different letters in the same column indicated significantly different (*p* ≤ 0.05).

**Table 2:** Thickness, water vaper permeability (WVP), and tensile strength (TS) of films.

Treatment	Thickness (mm)	WVP (g·mm/m^2^·day·kPa)	TS (MPa)
WG	0.22 ± 0.03 a	6.66 ± 0.32 b	6.70 ± 0.30 b
WG/PE0.5	0.20 ± 0.01 ab	7.46 ± 0.23 a	7.30 ± 0.60 b
WG/PE 0.75	0.18 ± 0.02 b	6.14 ± 0.18 bc	8.00 ± 0.00 a
WG/PE 1	0.19 ± 0.01 ab	5.06 ± 0.10 c	8.30 ± 0.30 a

Values are expressed as ave ± stdev. Different letters in the same column indicated significantly different (*p* ≤ 0.05). (Thickness, n = 5; WVP, TS, n = 3).

## Data Availability

The original contributions presented in the study are included in the article, further inquiries can be directed to the corresponding author.
